# Health care professionals’ attitudes regarding patient safety: cross-sectional survey

**DOI:** 10.1186/s13104-016-1977-7

**Published:** 2016-03-18

**Authors:** Indre Brasaite, Marja Kaunonen, Arvydas Martinkenas, Tarja Suominen

**Affiliations:** School of Health Sciences, University of Tampere, Tampere, Finland; Pirkanmaa Hospital District, Tampere, Finland; Faculty of Health Sciences, Klaipeda University, Klaipeda, Lithuania

**Keywords:** Attitude, Health care professionals, Nurses, Patient safety, Physicians, Nurse assistants

## Abstract

**Background:**

Patient safety is being seen as an increasingly important topic in the healthcare fields, and the rise in numbers of patient safety incidents poses a challenge for hospital management. In order to deal with the situation, it is important to know more about health care professionals’ attitudes regarding patient safety. This study looks to describe health care professionals’ attitudes regarding patient safety, and whether differences exist based on the background factors of study participants.

**Methods:**

A quantitative study using a questionnaire was conducted in three multi-disciplinary hospitals in Western Lithuania. Data was collected in 2014 from physicians, nurses and nurse assistants.

**Results:**

The results showed positive safety attitudes, and these were especially related to the respondents’ levels of job satisfaction. A respondent’s older age was associated with how they evaluated their teamwork climate, safety climate, job satisfaction, and perception of management. Profession, working unit, length of work experience, information received about patient safety during education, further education, and working shifts were all associated with several safety attitude areas.

**Conclusions:**

The safety attitudes of respondents were generally found to be positive. Attitudes related to patient safety issues were positive among health care professionals and opens the door for the open discussion of patient safety and adverse events. However, in future we also need to investigate the knowledge and skills professionals have in relation to patient safety, in order to gain a deeper understanding of the present situation.

## Background

Attitudes regarding safety-related issues are an important part of what is often called a hospital’s safety culture [1, 2]. An organization’s safety culture consists of components concerning healthcare provider attitudes about organizational factors such as safety climate and morale, work environment factors such as staffing levels and managerial support, team factors such as teamwork and supervision, and staff factors such as overconfidence and being overly self-assured [3]. Some authors [4–6] have noticed that a safety culture is a part of the wider organisational culture, and may be defined as the attitudes, beliefs, perceptions, competencies and values that determine an organisation’s health and safety management, and are held in common by employees in relation to safety. An understanding of nurses’ perceptions and expectations regarding adverse events is therefore essential for the implementation of appropriate strategies to manage nursing care. In this sense, the beliefs, values and organizational culture of registered nurses (RNs) are important aspects to be considered [7].

Ethical issues are integral to the topic of patient safety because it is known that millions of patients worldwide suffer injury or death every year as a result of unsafe medical practices and care, and patients are mostly harmed due to preventable causes that they receive during health care in hospital settings [8]. Health care professionals may know that their role is important in the delivery of safe care and that they should have positive safety attitudes. However, the results of a safety culture study [9] showed that both RNs and nurse managers were critical of the state of patient safety in acute care hospitals, with RNs being the more critical group. That said, generally positive attitudes to patient safety have been reported among health care professionals [10], and the safety climate within healthcare has been evaluated more positively by physicians than nurses [11].

Previous literature has shown some differences in attitudes regarding patient safety, based on profession, age, gender and working area. In one study, the connection of safety attitudes to profession was measured by researchers [12]. The results showed that only 39 % of physicians had a positive attitude towards safety climate, and less than half of the physicians and nurses surveyed were satisfied with their jobs (47 and 45 %, respectively). Physicians, nurses, and medical assistants had relatively similar but low perceptions of their working conditions when compared to managers (29, 36, and 35 %, respectively). Researchers have explored professional differences in patient safety attitudes among operating room (OR) care givers in nine medical centres [13]. Of the six patient safety domains covered in the study, stress recognition and working conditions showed significant differences by univariate analysis of profession. Regression analysis revealed that differences for job satisfaction and working conditions were seen among the professions studied. In intensive care units, surgeons have expressed more favourable perceptions of working conditions than nurses [14], and surgeons have also been seen to have a more favourable perception of management than OR nurses [13].

In a study conducted in the field of obstetrics, the highest positive safety attitudes score (48.3 %) was reported by the 30–35 years age group of health care professionals [15]. Associations between gender differences and patient safety attitudes are also to be found in the literature. Gender differences in patient safety attitudes were explored among OR care givers, and of the six patient safety scales, four showed significant differences in univariate analysis (teamwork climate, job satisfaction, perceptions of management, and working conditions). Women were found to have less favourable perceptions of teamwork [69, versus (vs) 76 for men], job satisfaction (74, vs 80 for men), management (60, vs 69 for men), and working conditions (57, vs 72 for men) [13].

Work area and discipline have also been reported to be associated with attitudes [4]. One of the key findings was that emergency department (ED) personnel, particularly ED nurses, perceived substantially lower levels of safety climate than workers in other areas. This suggests that the higher levels of risk and complexity, and the faster pace associated with work performed in emergency departments continue to require relatively more attention to be paid to safety issues than other areas.

To maintain a safe patient environment and safe practices, it is very important to promote the measurement and improvement of safety attitudes among health care professionals [16]. The research presented in this paper looks to describe health care professionals’ attitudes regarding patient safety, and whether differences exist based on the background factors of the study participants.

## Methods

### Data collection

The study was carried out in three hospitals in one region of Lithuania, and involved all of the health care professionals (physicians, head nurses, nurses and nurse assistants) who worked with adult patients. The hospitals involved are of similar size and provide multi-profile care for Western Lithuanian residents. The criteria for inclusion in the research were that participants were health care professionals, working in health care organizations (hospitals) with adult patients, and would participate voluntarily in the study.

Data was collected using a questionnaire consisting of background questions based on existing literature, and an instrument measuring patient safety attitudes. Twenty background questions investigated the basic demographic characteristics of participants (e.g. work position, place of work, age, gender, education, years at work, usual shift, working hours per week), as well as information concerning the type and hours of training they had received regarding patient safety. Finally, participants were asked how many adverse events they had reported during the previous year. They were also asked what kind of patient safety related events they had faced, and whether they had reported them.

The data for measuring safety attitudes was collected using the University of Texas safety attitudes questionnaire (SAQ) [3] (short form version) that consists of six scales: teamwork climate, safety climate, perceptions of management, job satisfaction, working conditions, and stress recognition. Additional to the SAQ, five further statements examining safety attitudes were included, such as the health care professionals’ perceptions of whether safety issues would be acted upon if they expressed them to management, and whether they experienced good collaboration with other nurses, staff physicians and pharmacists in their clinical area. A final statement examined if communication breakdowns that lead to delays in the delivery of care were common. The SAQ (short form version) used in this study comprised of 36 items, each answered using a six-point Likert scale: 1 = disagree strongly, 2 = disagree slightly, 3 = neutral, 4 = agree slightly, 5 = agree strongly, and 6 = not applicable. Negatively worded items were reverse scored so that their valence matched the positively worded items.

The SAQ (short form version) was used because of its usability, the good psychometric properties it has shown in previous studies, and its broad potential for implementation [3, 17]. The instrument was originally developed in the United States of America and was translated from English into Lithuanian using the back-translation technique [18]. Permission to use the instrument in this study was obtained from the copyright holder of the instrument by the one of the authors. The questionnaire was piloted in one regional hospital with health care professionals to evaluate the validity of the instrument, and also its use in the Lithuanian context. The pilot data collection took place in February 2014, and included the hospital staff (n = 90). The pilot study hospital was not included in the main study. The SAQ showed good psychometric properties. The scales reliability was assessed with a total Cronbach’s alpha of .78, corrected by inter-item correlation from .05 to .69. The Cronbach’s alpha values were good for all scales for the main (and pilot) study: for teamwork climate .62 (.66), safety climate .74 (.78), job satisfaction .87 (.86), stress recognition .79 (.88), perceptions of management .90 (.92), and Working Conditions .74 (.78).

Ethical approval for the study was obtained from the Ethics Committee of Klaipeda University, Faculty of Health Sciences (Nr. 46 Sv-SL-1), and permission to collect data was also obtained from the hospitals participating in the pilot and main phases of the study. The ethical considerations related to the data collection were focused on the ethical principles for research, namely confidentiality (related to questionnaires), privacy, and the voluntary nature of participation in the study [19].

The main study data was collected in May 2014 in three regional hospitals. In each hospital, questionnaires with return envelopes were delivered to established contact persons. The contact persons circulated the questionnaires to all staff (n = 1687). After 2 weeks, the researcher collected the returned questionnaires in sealed envelopes from each unit. Because of a low response rate [46 % (n = 774)], reminder letters were left for the contact persons who were asked to circulate them. An additional 2 weeks were given to respond, and the final response rate was 64 % (n = 1082).

### Statistical analyses

Descriptive statistics were used to describe the characteristics of respondents (physicians, head nurses, nurses and nurse assistants), the SAQ items, and the scale-level results of the three hospitals. Differences in sample characteristics between hospitals and professional groups were tested using one-way analysis of variance (ANOVA) and the Tukey HSD (honest significant difference) multiple comparison test, or the Tamhane multiple comparison test (when the assumption of equal variances was not correct). Non-normally distributed characteristics were analysed using the Kruskal–Wallis test. Data was presented using mean [standard deviation (SD)] or median [interquartile range (IQR)] expression. Any negatively worded items of the SAQ were reversed before analysis. The internal consistency of the SAQ and its scales of safety climate, job satisfaction, perception of management, and working conditions (for SAQ) was measured by calculating the Cronbach’s alpha for each area. Associations between variables were calculated by means of Spearman correlations.

For further analysis, the units in which the respondents worked were re-grouped as internal medicine (e.g. internal diseases, neurology, cardiology, heart arrhythmia, haemodialysis, nephrology etc.), surgical (e.g. surgery, traumatology), psychiatric (e.g. mental health, treatment of addiction), acute (e.g. resuscitation, anaesthesiology, emergency, operating room, intensive care), and others (e.g. rehabilitation, laboratory, polyclinics etc.). Head nurses and nurses were also combined into one group (756 nurses including 54 head nurses). All of the data was analyzed using SPSS (Statistical package for social sciences) (version 22.0; SPSS Inc., Chicago, Illinois, USA). A *p* value of <.05 was considered to be statistically significant.

## Results

### Participants

The questionnaire was answered by 1082 (64 %) of the health care professionals surveyed. Participants were nurses (n = 756, 70 %), nurse assistants (n = 180, 16.6 %) and physicians (n = 146, 13.5 %). Most participants were female 989 (91.4 %) and their mother tongue was Lithuanian 1018 (94.1 %). The most common education institutions of the study participants were medical school 493 (45.6 %), college 130 (12.0 %), and a university bachelor programme 118 (10.9 %). Respondents stemmed from three regional hospitals: 301 (27.8 %) from hospital 1, 411 (38.0 %) from hospital 2, and 370 (34.2 %) from hospital 3. The mean age of the participants was 46.7 years (SD = 10.9). Most had a permanent position at their hospital (n = 1047, 96.8 %), the mean work experience was more than 20 years (mean = 23.9), and they worked an average of 39.9 h per week in their unit. Some health care professionals (n = 140, 12.9 %) had an extra job and worked an average of 18.6 h per week in this setting. Most of the health care professionals (n = 659, 60.9 %) worked variable shifts, and in units with averages of 30.7 beds and 24.9 staff members. Usually, one health care professional had 18 patients per working shift. Almost two thirds of the participants (n = 673, 62.2 %) had received no information about patient safety during their initial professional education, but about half (n = 589, 54.4 %) had received some in their further education (Table 1). 80 % of respondents (n = 866) had not reported any patient safety incidents during the last year.

**Table 1:** Work related background factors (n = 1082)

	Mean (SD)	Median (IQR)
Years of experience in primary speciality	21.61 (12.04)	22.00 (17)
Years of work experience in general	23.88 (11.52)	25.00 (15)
Years worked in this unit	14.32 (11.80)	12.00 (15)
Working hours per week in this unit	39.86 (8.23)	38.00 (2)
Hours per week in extra job	18.61 (14.63)	16.50 (16)
Number of beds per unit	30.72 (17.27)	30.00 (20)
Total number of staff working in unit	24.09 (10.33)	23.00 (10)
Number of patients health care professionals usually have per working shift	18.00 (12.03)	15.00 (12)
Health care professionals working in unit		
Day shift		
Physicians	4.28 (3.15)	3.00 (4)
Nurses	4.72 (4.82)	3.00 (3)
Nurse assistants	2.48 (2.14)	2.00 (2)
Evening shift		
Physicians	1.69 (1.47)	1.00 (1)
Nurses	2.34 (1.81)	2.00 (1)
Nurse assistants	1.62 (1.08)	1.00 (1)
Night shift		
Physicians	1.19 (.82)	1.00 (0)
Nurses	1.90 (1.15)	2.00 (1)
Nurse assistants	1.28 (.67)	1.00 (0)
Usual shift	N (%)	
Day	398 (36.8)	
Evening	2 (.2)	
Night	7 (.7)	
Variable shifts	659 (60.9)	
Other	14 (1.3)	
Extra job		
Yes	140 (12.9)	
No	939 (86.8)	
Information about patient safety during initial education		
Yes	408 (37.7)	
No	673 (62.2)	
If yes, hours [mean (SD), median (IQR)]	37.14 (58.49)	20.00 (30)
Information about patient safety in continuing education		
Yes	589 (54.4)	
No	492 (45.5)	
If yes, hours [mean (SD), median (IQR)]	24.22 (32.02)	14.00 (30)

### Safety attitudes

The results of this study show positive safety attitudes overall, in regard to job satisfaction (mean = 4.14), teamwork and safety climate (mean = 4.10 in each domain), and working conditions (mean = 4.09) (Table 2). Only in the area of perceptions of management there were differences (p < .001) to be seen between the three hospitals participating in the study.

**Table 2:** Patient safety attitudes

SAQ Short form scales/hospitals	Mean (SD)	Median (IQR)	Chi square	p value
Teamwork climate				
Hospital 1	4.07 (.64)*	4.07 (.64)*		
Hospital 2	4.08 (.72)	4.08 (.72)		
Hospital 3	4.16 (.67)*	4.16 (.67)*		
Total	4.10 (.68)	4.10 (.68)	3.84	.147
Safety climate				
Hospital 1	4.07 (.67)	4.07 (.67)*		
Hospital 2	4.05 (.72)^‡^	4.05 (.72)^‡^		
Hospital 3	4.17 (.67)^‡^	4.17 (.67)*,^ ‡^		
Total	4.10 (.69)	4.10 (.69)	7.86	.020
Job satisfaction				
Hospital 1	4.21 (.84)*	4.21 (.84)*		
Hospital 2	4.05 (.90)*	4.05 (.90)*, ‡		
Hospital 3	4.19 (.84)	4.19 (.84)^‡^		
Total	4.14 (.87)	4.14 (.87)	6.35	.042
Stress recognition				
Hospital 1	3.86 (.88)	3.86 (.88)		
Hospital 2	3.79 (.94)	3.79 (.94)		
Hospital 3	3.75 (1.01)	3.75 (1.01)		
Total	3.80 (.95)	3.80 (.95)	1.12	.572
Perceptions of management				
Hospital 1	3.75 (.83)*	3.75 (.83)*		
Hospital 2	3.58 (.89)*,^ ‡^	3.58 (.89)*,^ ‡^		
Hospital 3	3.83 (.88)^‡^	3.83 (.88)^‡^		
Total	3.71 (.88)	3.71 (.88)	20.76	<.001
Working conditions				
Hospital 1	4.05 (.97)	4.05 (.97)		
Hospital 2	4.04 (.98)	4.04 (.98)^‡^		
Hospital 3	4.18 (.96)	4.18 (.96)^‡^		
Total	4.09 (.97)	4.09 (.97)	5.54	.063

For Means: ANOVA + Tukey HSD multiple comparison test

For Ranks: Kruskal–Wallis Test + Mann–Whitney U comparison test

* p < .05—differences between Hospital 1 and Hospital 2 or Hospital 3

^‡^ p < .05—differences between Hospital 2 and Hospital 3

The most positive safety attitudes represented in the SAQ scales tended to correlate with the most background factors, namely safety climate, job satisfaction, perceptions of management and working conditions. Older health care professionals were associated with how they evaluated teamwork climate (.061), safety climate (.078), their job satisfaction (.150) and their perceptions of management (.140). The length of work experience in general was associated with how participants evaluated their safety climate (.082), job satisfaction (.155) and their perceptions of management (.193). Respondents who had received information about patient safety during their education were associated with how they reported their teamwork climate (−.090), safety climate (−.093), job satisfaction (−.076), perceptions of management (−.093) and working conditions (−.072). Those who had received information about patient safety in continuing education reported the same associations, with the exception of teamwork climate. Whether the health care professional worked day shifts or variable shifts was associated with her/his safety attitudes in all of the investigated safety areas: teamwork climate (−.108), safety climate (−.089), job satisfaction (−.137), stress recognition (−.088), perceptions of management (−.188) and working conditions (−.154) (Table 3).

**Table 3:** Correlations between respondents’ background factors and their patient safety attitudes

Demographic characteristics	Teamwork climate	Safety climate	Job satisfaction	Stress recognition	Perceptions of management	Working conditions
Age	.061*	.078*	.150*	−.013	.140*	.053
Gender	−.041	.013	−.017	−.083*	−.029	.039
Education	.101**	.024	.003	.059	.090**	.044
Years of experience in primary speciality	.007	.041	.086**	−.003	.036	.046
Years of work experience in general	.053	.082**	.155**	−.033	.093**	.053
Information about patient safety during initial education	−.090**	−.093**	−.076*	−.007	−.093**	−.072*
Information about patient safety in continuing education	−.058	−.111**	−.100**	.018	−.099**	−.063*
Received hours regarding information about patient safety in continuing education	−.052	.005	−.017	−.039	.015	−.146*
Usual shift	−.108**	−.089**	−.137**	−.088**	−.188**	−.154**
Working hours per week in this unit	−.040	−.026	−.054	.067*	−.059	−.049
Extra job	.027	.081**	.047	−.046	.014	.025
Number of beds per unit	−.038	−.074*	−.066	.056	−.039	.003
Total number of staff working in unit	−.040	−.006	−.009	.079*	−.092**	−.057
Number of physicians working in unit on day shifts	−.024	.042	.046	.039	.056	.110**
Number of nurses working in unit on day shifts	.036	.034	.091**	.005	.046	.081*
Number of patients health care professionals usually have per working shift	−.020	−.033	−.095**	−.012	−.053	−.078*

Spearman correlations, * p < .05, ** p < .01

Physicians had significantly higher safety attitudes related to teamwork climate (p = .014) and Stress Recognition (p < .001) than nurses and nurse assistants in the group of health care professionals who did not report a safety incident during the last year, but the attitudes towards the Perceptions of Management (p < .001) in the same group were significantly higher for physicians and nurse assistants than nurses. In the health care professional group who had reported a safety incident during last year, physicians had significantly higher safety attitudes related to their teamwork climate than nurses and nurse assistants (p = .011). Those who didn’t report any safety incidents during the last year had more positive attitudes towards Stress Recognition than those who had reported such incidents.

When comparing safety attitudes between health care professionals by working unit, some significance differences were noted. Health care professionals working in psychiatric units had significantly lower safety attitudes relating to job satisfaction (p = .004), perceptions of management (p < .001) and working conditions (p < .001) than those working in internal medicine, surgical, acute or other units.

Nearly two thirds of health care professionals (n = 638, 59 %) felt that their suggestions about safety would be acted upon if they expressed them to management, whilst 20.1 % (n = 218) were neutral and 18.1 % (n = 195) disagreed. Most of the respondents experienced good collaboration with nurses (n = 914, 84.5 %), with staff physicians (n = 859, 79.4 %), but less with pharmacists (n = 248, 22.9 %) in their clinical area. Only 18.7 % (n = 203) of health care professionals felt that communication breakdowns that lead to delays in the delivery of care were common.

## Discussion

Our goal was to assess the general situation regarding the safety attitudes of health care professionals, because no national-level data had been reported in either Lithuania or any of the other Baltic Countries. Overall, the safety attitudes of health care professionals were positive and in-line with previous studies (e.g. [10, 20–22]). However, whilst the results of this study were partly in-line with earlier results, there were also contradictory elements. As such, further study is needed to establish links between these areas, and the attitudes and background factors of individual respondents, and this may prove important in developing our clinical practices. Age seemed to be associated with many safety attitudes scales, and it has previously been reported [15] that the highest positive safety score when comparing younger and older age groups was to be found to be in the 30–35 year age group. In our study however, safety attitudes were found to be higher in older age groups. This may be explained by the linked years of work experience (mean = 23.9) which indicates that health care professionals who know their job very well, may also hold enhanced safety attitudes.

Gender was only associated with stress recognition, although a previous study [13] has shown gender to be associated with several safety attitudes such as teamwork climate, job satisfaction, perceptions of management, and working conditions. In our study, only about 10 % of the respondents were male, which may have had an effect on the results.

It was interesting to find that physicians had higher safety attitudes towards teamwork climate than nurses and nurse assistants. this may indicate that physicians tend to value teamwork more when adverse events happen, and perhaps consider the issue to be faced as more of a common responsibility than other health care professional groups. Our result is similar to previous positive physician safety attitudes reported by other researchers, for example where physicians had more positive attitudes in perceptions of their working conditions [14], and in their perceptions of management [13] than nurses.

In comparing safety attitudes between health care professionals by work area, it was found that respondents in psychiatric units had significantly lower safety attitudes than those working in internal medicine, surgical, acute and other units. This might be linked to their working environment, as health care professionals may be more stressed when working with patients with mental illnesses, and sometimes be subjected to physical or psychological violence from their patients. Another explanation may be that health care professionals think of safety issues differently depending upon the type of treatment involved (e.g. operations, infections, or patient falls), and some of these issues may not be seen to be so relevant in psychiatric units.

Other researchers [4] have also highlighted differences in attitudes between work areas, with the main finding that ED personnel perceived substantially lower levels of safety climate than workers in other clinical areas.

Health care professionals who received no information about patient safety during their initial professional education had more negative attitudes to teamwork climate, safety climate, job satisfaction, perceptions of management and working conditions than those who had. Also, health care professionals who received no information about patient safety during their further/continued education had lower ranked attitudes to safety climate, job satisfaction, perceptions of management and working conditions than whose who had. Drawing from this, we may consider that education about patient safety impacts upon the safety attitudes of health care professionals.

### Methodological considerations

There are several strengths in this study. Especially, the response rate was good (64 %) and we were able to reach both nurses and physicians in the same study. A limitation may be noted in that the data was purposefully collected from one region. However, we consider these results to present a fairly representative view of patient safety attitudes that may be predicted in similar size multi-profile hospitals across Lithuania, as the country is divided into 10 similar size regions where health care services are organized using the same structure, and serve similar sized populations. Thus the results may readily transpose to a wider setting within Lithuania, but they may not be representative of other national settings.

## Conclusions

Attitudes related to patient safety issues are positive among health care professionals in Lithuania, which helps to open the door for the open discussion of patient safety and adverse events. However, in future we also need to investigate the knowledge and skills professionals have in relation to patient safety, in order to gain a deeper understanding of the present situation.

## Abbreviations

RNs: registered nurses
OR: operating room
VS: versus
ED: emergency department
SAQ: safety attitudes questionnaire
ANOVA: one-way analysis of variance
HSD: honest significant difference
SD: standard deviation
IQR: interquartile range
SPSS: statistical package for social sciences
